# Baltimore Academy of Medicine

**Published:** 1878-07

**Authors:** 


### Reports of Societies.

### MEETING OF THE BALTIMORE ACADEMY OF MEDICINE, APRIL 16TH, 1878.

Dr. Wm. Lee related the case of a lady suffering with severe gastric pains of a neuralgic character, accompanied by nausea, vomiting, headache, and exhaustion, in which the symptoms were relieved by injecting subcutaneously the hydrobromate of quinia, in m. xx, twice daily for six days. This case had lasted several weeks, and resisted the usual remedies.

Dr. Chisolm related some cases illustrating the inappropriate treatment often resorted to in eye diseases.

1. A patient who was cut through the cornea by the glancing of a nail, with emptying of the aqueous chamber and a protrusion through the wound of a portion of the iris, forming an iritic hernia, was treated by poultices and blue pills, followed by heavy saline purgatives, which treatment was kept up persistently for a week, at which time the case was sent to him by the family physician. Cutting off the protruding iris, and liberal use of atropia very soon brought about improvement.

2. A lady suffering under specific iritis, innocently acquired, was actively treated by a strong nitrate of silver collyrium, much to her detriment. This, apparently, is the stereotyped treatment of many practitioners, who mistake the conjunctival injection for the primary lesion, and neglect to use the atropia drop, which, in all cases, will clear up the diagnosis, and under no condition can do harm.

Dr. Chisolm also related the following odd case of injury to the eye:

A child eighteen months old fell upon the floor and got up screaming, with his hand to his eye. A casual examination of the eye indicated no injury, but as the child continued to cry, and to direct attention to the eye, further examination was made, and a glistening metallic point was found protruding in the vicinity of the caruncula, which being drawn upon, an entire pin was extracted. It must have been sticking upright in the floor, when the child falling upon it, drove it headlong into the socket, between the eyeball and lachrymal sac.

In connection with this, Dr. C. related a case, formerly reported by him, in which a girl, whilst shaking a carpet, received, as she thought, some foreign body in the eye. Upon careful examination, the head of a pin was seen sticking to the upper lid, and when pulled upon the pin was drawn out entire. Driven with force by the shaking of the carpet, it had entered the upper eyelid point foremost, and its velocity was sufficient to drive it entirely through the eyeball, the pin-head alone preventing its disappearance in the socket. The point had escaped at the fovea centralis, and had destroyed vision by causing, in course of time, detachment of the retina.

Dr. Erich reported the case of a girl aged seventeen, in whom the vagina was absent. The uterus was present, and for four years she has suffered terribly every month during her menstrual periods, requiring large doses of morphia for her relief. A surgeon in the city once attempted an operation upon her, but desisted after penetrating the rectum. This aperture has healed up, and Dr. E. proposes to attempt to make a passage with the knife through the fibrous tissue to the uterus.

Dr. McKew reported a case of cystic trouble, characterized by inability to retain the urine longer than from one-half to one hour, and the urine passed being sometimes bloody. No calculus could be discovered, and kidneys and urethra were healthy. Drugs having been used ad nauseum, milk diet was advised; this was begun last November, and kept up strictly to the present time. The patient expresses himself as feeling "very comfortable" under this limited diet, and says he does not know what it is to feel either hunger or thirst. At first the bowels were constipated, but afterwards became regular. He still passes urine very frequently, but can retain it two or three hours, and he is very well satisfied with this result. He presents a hearty and robust appearance.

Dr. McKew also related a case of otitis media, in which the purulent discharge takes place through the nose and mouth, as well as the meatus auditorius externus. When the patient holds his head over and presses on the healthy side, pus flows from the diseased ear.

Dr. Chisolm said it was not uncommon for the discharge to flow out through the nostrils, since it finds its way into the pharynx through the eustachian tube, and is thence directed along a sort of slight ridge into the posterior nares. He also said that in case of rupture of the membrana tympani, injections into the nares will emerge from the external auditory meatus; indeed, this is the method of treatment employed in affections of the middle ear, such as Dr. McKew has described. The patient is made to swallow at the moment the injection is made, by which the palate is raised to a level with the floor of the posterior nares, and the walls of the pharynx compressed, the fluid by these means being forced to make its way into the eustachian tubes, thence to the middle ear. In using the nasal douche, it is therefore necessary to be cautious when strong injections are employed. A eustachian catheter is not essential in the injections referred to; indeed, they cannot be made through it. The explanation of the effect of pressure in causing the discharge of pus from the ear, is probably to be found in the fact that the purulent collection is subpericranial.

Dr. J. Carey Thomas reported that he had been using glycerine in drachm doses, thrice daily, in several cases of piles with favorable results. It produces a slightly laxative effect, also soothes and relieves pain.

Dr. McKew referred to a case of diabetes mellitus (with a s. g. of 10 36, and urine heavily laden with sugar), in which the thirst was relieved by the muriated tincture of iron.

Dr. Erich had found irritability of the bladder one of the most troublesome symptoms in diabetes; in one case the patient was unable to retain his urine longer than one hour.

Dr. McKew reported that his experience with dialyzed iron is unfavorable.

Dr. Chew said that he finds it as useful and reliable as any of the chalybeates, and thinks it likely to prove as effective an antidote for arsenious acid as the sesqui-oxide of iron.

Dr. Arnold prefers the older preparations, as iron by hydrogen, and the pills recommended by Niemeyer. There is an unofficial preparation (tinctura ferri pomata) kept in the German shops in this city, which he finds a reliable chalybeate; it is made by mixing iron and mashed apples, the result being a malate of iron. He believes the undoubtedly beneficial effects of muriated tincture of iron in erysipelas due not to the iron, but to the muriatic acid, and he finds the latter used alone equally as efficacious as the former.

Dr. Arnold related a case of otorrhœa, in a man of forty, which had lasted from childhood. The patient was subject to three or four times a year to attacks of acute mania, each lasting about six weeks. Thinking these might be due to the discharge from the ear, he sent the case to an aurist, who cured the otorrhœa, since which (nine months) he has had no occurrence of the mania.

---

Baltimore, Md., May 7th, 1878.

Dr. Cordell reported the case of a real estate agent, aged fifty-eight, height 6 feet 3½ inches, weight 120 pounds. At the age of fourteen he had typhoid fever; since that time has never been sick. Weight is the same now as at twenty-one. At the age of twenty-five he lost his appetite for breakfast, and has ever since dispensed with that meal. At the ago of fifty-two, owing to the distance from his place of business to his residence, he gave up dinner also; for the last six years he has, therefore, confined himself to one meal a day, and that about 6 p.m. From the time of rising in the morning until this hour, not a particle of food or fluid of any kind enters his mouth. During the six years in which he has partaken of but the one daily meal, his health has been excellent, he has had no dyspepsia, and his bowels have been open once daily. He uses no stimulants, and for the last two years has drunk no tea nor coffee. The evening meal is moderate in quantity, embracing the usual variety of a dinner table; he rarely, however, eats any other meats but fresh pork and bacon. Between the meal and bed-time he drinks a large quantity of water.

Dr. Richard McSherry reported the case of a lady with mitral insufficiency and regurgitation, who two weeks ago had a congestive chill; the pulse was imperceptible, and she occasionally coughed up bloody mucus. Whisky administered by the mouth was immediately ejected. The prognosis was exceedingly unfavorable. The treatment consisted in cautiously allowing the inhalation of ether, rubbing the surface with ammonia, and applying sinapisms, and in injecting hypodermically equal parts of whisky and ether at frequent intervals. In eight hours she became conscious and was able to speak. The patient's life was, he believed, saved by the hypodermic treatment.

Dr. McKew referred to a case in the practice of a prominent physician in Washington, in which fatal narcosis resulted from a hypodermic injection of grain one-third of morphia in pneumonia. He (Dr. McK.) begins generally with grain one-fifth. Has never seen any ill-effects from such treatment.

Dr. McSherry referred to a case of pleuritis, occurring many years ago under his care, in which fatal narcosis resulted from morphia administered per orem, in repeated small doses, the amount taken in all not exceeding gr. i. On *post mortem* examination, the pleural sac on one side was found filled with gelatinous matter, which had strongly compressed the lung on that side.

Dr. Chisolm had seen grs. iij, given hypodermically in one habituated to the use of the drug in this manner, and once met at his office a newspaper reporter, addicted to opium eating, who told him that he had taken ʒ ij at one dose, with suicidal intent, without any bad effect. He also had a patient aged seventy, one of the best legal minds in the section of country in which he lived, who took daily grs. xij, and still continued the habit without detriment. He had also seen another case in which ʒj was taken at a dose.

Dr. Ward had seen a patient, a boy with rheumatism, who took by mistake gr. iss of morphine, with no ill effect, but decided benefit, the rheumatism being entirely relieved.

Dr. McKew had seen a patient who took about ʒj at a dose.

Dr. Erich regulates the dose according to the amount of pain, and with a person accustomed to the use of tobacco and other stimulants, he begins with gr. ss. A patient of his with asthma, took ʒss. of morphia, which produced contraction of the pupils and stupor. These symptoms were relieved by electricity. He also used chloroform and ergot hypodermically, and once saved a patient suffering with post partem hemorrhage, by subcutaneous injections of brandy. He has never seen an abscess result from hypodermic injections of morphia, which he attributes to the fact that as soon as cloudiness appears in his solution, he ceases to use it, or at least filters it through his handkerchief, which can easily be done at the moment.

Dr. McKew said that he also had never had an abscess follow his injections, although he paid no attention to the cloudiness of the liquid; he does not regard as the cause of abscesses the minute organisms upon which the opacity depends.

Dr. Lee had been informed that in Paris they seek to avoid after-trouble by using cherry-laurel water; he adds to his solution a minute quantity of carbolic acid. He also referred to the treatment of anæmia by hypodermic injections of solution of dialyzed iron.

Dr. Chisolm quoted statistics of McCarthy Dawson, of London, showing the fatality of ether inhalations; in the report 151 deaths from ether had been collected.

Dr. H. P. C. Wilson regards ether equally as dangerous as chloroform.

Dr. Chisolm reported several cases of eye and ear trouble, as follows:

1. Rupture of membrana tympani from a person suddenly and gently pressing his open palms over the ears of the patient from behind. The rupture was due to the compression of the air in the meatus auditorious externus. Buzzing, dizziness, and slight dullness of hearing resulted immediately. The prognosis of such cases depends upon whether the bones of the tympanum have been displaced inwards into the labarynth or not; in the former case the loss of hearing and vertigo will be permanent, in the latter complete recovery will take place. The eustachian tube is closed except in gaping and yawning; hence the air cannot make its escape by that channel.

2. Case of supposed amaurosis in a child four years of age, presenting a spot in the centre of the pupil, due to intra-uterine ulceration of the cornea, and escape of aqueous humor. On further examination, double cataract was also found, zorial in character, but of extensive development, which, as it was surrounded by a layer of healthy lens substance, gave a blackness to the pupil quite different from the usual white opacities of the lens in childhood. It was only under full dilatation of the pupil fıom atropia, that the red reflex of choroid could be perceived around the margins of the lens.

3. Case of ciliary staphyloma of long standing, with complete destruction of vision, exciting sympathetic ophthalmia in the other eye. A blow from a piece of stick in cutting wood had struck the staphylomatous projection, and ruptured the eyeball, necessitating enucleation.

4. Case in which an explosion of a keg of gunpowder took place, twenty-three years ago, injuring the face extensively, especially the forehead. During the cicatrization from the sloughing, the upper lids were absorbed by contraction of the eyebrows, causing marked ectropion and exposure of the eyeballs. To relieve this condition, the lash border was loosened from its elevated position, drawn down to its proper place, and a tongue of skin cut away from the temple, was inserted for the formation of a new lid. As the mucous membrane still pouted in excess, it was necessary to restore the upper *cul-de-sac* by the application of a suture support, passing from the brow downward. Final result of case very satisfactory. In this connection Dr. C. reported several cases of blepharoplasty, in which the new lids had been taken from various parts of the face, sometimes from the skin of the temple as in this instance, at other times a plug of skin had been taken from the cheek with footstalk from the temporal region; again a flap of skin from a direction parallel with nose, with footstalk at inner canthus. The only objection to temporal flaps was the free hemorrhage from cutting the temporal artery.

5. Case illustrating slowness of development of cataract. Patient seventy odd years of age. Saw the patient first in 1868; found then incipient cataract. A drawing of the striations was then made. A comparison of this drawing with the appearance now presented shows that there has been no change after nine years, although the prospect from general experience was that in 18 months or two years the cataract would have occupied the whole of the lens.

6. Case illustrating the rapid development of cataract.

A patient had incipient cataract, but had good sight for two years, until one morning on rising he found his sight gone.

Dr. Erich brought up the subject of the comparative fatality of craniotomy and Cæsarean section. The former is far less serious than the latter; statistics show twenty-five per cent. of deaths in Cæsarean section. He has had fifteen cases of craniotomy without a fatal result. Every effort was first made in these to extract with the forceps; one of the fifteen was an eleven months fœtus, the nails were long, and it was as large as an infant of two months, whilst the mother was a woman of small size. Another case was a twin-pregnancy, in which craniotomy was performed upon the first child after unsuccessful use of the forceps; the forceps also failed with the second, which was delivered dead-born after the performance of version. His rule is, when forceps have been tried in vain, to proceed at once to craniotomy, not to attempt version. He referred to a case in which a surgeon operated with Smellie's scissors; the uterus was perforated, the bowels protroded through the rent, and were drawn out by the operator, who then recognized what they were and restored them as best he could, but the patient died. We must give due weight to the consideration that craniotomy is done by all practitioners; the Cæsarean section by surgeons only.

Dr. McKew said that the Cæsarean section had not been performed sufficiently often in this city to decide the question proposed by Dr. Erich. He has had three cases of craniotomy, none of Cæsarean section on the living subject. Craniotomy done at the proper time does not compromise the tissues of the mother. Has never seen any ill results from craniotomy in his own or the practice of others.

Dr. H. P. C. Wilson has performed craniotomy three times, and agrees with Dr. Erich as to the safety of the operation when performed at the proper time.

Dr. Van Bibber said the operation of craniotomy should, as a rule, be limited to those cases in which the diameter of the pelvis is less than three inches. He related a case of extra-uterine fœtation, in which, after lasting several days, the pains subsided, and the patient lost sight of. Six years and a half afterwards, she came under the care of Dr. C. Johnston, suffering with symptoms of chronic dysentery. He was led by peculiarities of the case, to make a digital examination of the rectum, and thus detecting the presence of a foreign body, he extracted portions of the skull of the fœtus, and finally the entire remains. He also related a case in which the pelvic canal was so encroached upon by an osseous tumor, that not only was natural labor prevented, but the Cæsarean section was seriously contemplated. In the emergency, it was determined to try and deliver the fœtus by drawing down the extremities. This was successfully done, and the body extracted, but the head could not be gotten out, and the woman finally died exhausted. A consideration of these cases had suggested to him whether it were not better to leave the child undisturbed in utero, and trust to the resources of nature, in these dystociæ so seriously jeopardizing the life of the mother.

Dr. Erich thought the patient would certainly perish from septicæmia if this course were adopted.

Dr. McSherry preferred Cæsarean section to craniotomy in all cases where the child is living.

Dr. McKew thought craniotomy should not be performed upon a living fœtus; we have no right to destroy a life to save a life.

Dr. J. Carey Thomas reported a case in which the end of a vaginal syringe was broken off in the vagina, and passed afterwards with the menstrual flow.

Dr. Van Bibber reported the case of a lady at the "Carrollton," on her wedding trip, from whose vagina he extracted three pieces of broken syringe.—*Maryland Medical Journal.*

